# A24 INVESTIGATING THE IMPACT OF PARKINSON’S DISEASE-ASSOCIATED GENES ON INTESTINAL HOMEOSTASIS

**DOI:** 10.1093/jcag/gwac036.024

**Published:** 2023-03-07

**Authors:** J Pei, S J Recinto, A MacDonald, C Gavino, L -E Trudeau, M Desjardins, J A Stratton, S Gruenheid

**Affiliations:** 1 Microbiology and Immunology; 2 Integrated Program in Neuroscience, McGill University; 3 Département de neurosciences; 4 Département de pathologie et biologie cellulaire, Université de Montréal , Montreal, Canada

## Abstract

**Background:**

Intestinal epithelial cells (IECs) provide an essential physical barrier between harsh luminal contents and underlying host tissue. The maintenance of intestinal homeostasis in this rapidly renewing tissue must be intricately regulated through the proliferation and differentiation of intestinal stem cells (ISCs). Dysregulation of this system results in the loss of barrier function, causing pathologies in both intestinal and extra-intestinal diseases. While Parkinson’s Disease (PD) is primarily a neurodegenerative disorder, there is increasing evidence linking PD progression and gastrointestinal dysfunction. For instance, constipation and increased bowel permeability are frequently observed years prior to development of motor dysfunction in PD, people with inflammatory bowel disease are more likely to develop PD, and a positive correlation exists between gastrointestinal infections and PD incidence. Our group recently developed a model to investigate the role of the gut in PD, demonstrating that mice with genetic ablation of the PD-associated gene *Pink1* exhibited motor phenotypes only when previously infected with Gram-negative *Citrobacter rodentium* intestinal bacteria. As *Pink1* and other PD-associated genes are expressed in IECs, we hypothesize that PD-associated gene mutations directly affect the epithelium and impact early PD pathophysiology.

**Purpose:**

Investigate the impact of *Pink1* and other PD-associated genes in IECs under steady state and infection.

**Method:**

Single-cell RNA sequencing was performed on IECs isolated from *Pink1* WT and KO mice, at steady state and following *in vivo C. rodentium* infection. Mice were sacrificed at an early timepoint of infection (day 6) to elucidate transcriptional differences between epithelial lineages of each genotype. Additionally, *ex vivo* colonoids were derived from primary mouse tissue and treated with lipopolysaccharide (LPS) to determine how PINK1 loss-of-function affects the inflammatory response of the epithelium.

**Result(s):**

Our data revealed that loss-of-function of PINK1 profoundly affected the ISC compartment and several epithelial lineages. Specifically, ISCs from infected *Pink1* KO mice demonstrated differentially regulated proliferative and cell cycle genes, while transit amplifying cells showed dysregulated expression of tight junction genes, and enterocytes displayed differentially expressed oxidative damage and apoptotic genes. Preliminary data from colonoids showed that *Pink1* KO mice, when stimulated with LPS, had increased pro-inflammatory cytokine gene expression.

**Conclusion(s):**

In *Pink1* KO intestinal epithelial cells, there is indeed an altered cellular response upon infection *in vivo* and LPS treatment *ex vivo*. However, more information is needed to decern the mechanistic role of IECs in PD. By investigating the role of PD genes in the gastrointestinal tract, these studies carry important implications for understanding the initiation and progression of PD.

**Disclosure of Interest:**

None Declared

